# Developing, Implementing, and Evaluating a Multimedia Patient Decision Aid Program to Reform the Informed Consent Process of a Peripherally Inserted Central Venous Catheter Procedure: Protocol for Quality Improvement 

**DOI:** 10.2196/10709

**Published:** 2018-12-18

**Authors:** Azizeh Khaled Sowan, Arlienita R Beraya, Adrian Carrola, Charles C Reed, Sherrie V Matthews, Tarryn Moodley

**Affiliations:** 1 School of Nursing University fo Texas Health at San Antonio San Antonio, TX United States; 2 Center for Clinical Excellence University Health System San Antonio, TX United States; 3 Transplant Cardiac ICU University Health System San Antonio, TX United States; 4 Corporate Communications & Marketing University Health System San Antonio, TX United States; 5 Clinical Systems University Health System San Antonio, TX United States

**Keywords:** multimedia, central venous catheters, decision support techniques, informed consent, intensive care units

## Abstract

**Background:**

Informed consent has considerable clinical, ethical, and legal implications for patient safety and liability. Little information is available about the use of multimedia patient decision aids (PtDA) in the consent process for therapeutic invasive procedures such as the peripherally inserted central venous catheter (PICC). In addition, none of the available studies have designed their multimedia PtDAs based on the Agency for Healthcare Research and Quality’s (AHRQ) comprehensive guide for informed consent.

**Objective:**

This paper describes a patient-centered, systematic, multidisciplinary approach to develop, implement, and *alpha* test a multimedia PtDA to reform the informed consent process of a PICC for patients in 10 acute and intensive care units.

**Methods:**

The development, implementation, and evaluation processes of the PtDA followed the phases in the Multimedia Production Framework: preproduction, production, and postproduction. Within this framework, we applied the criteria for judging the quality of PtDAs, the AHRQ’s Health Literacy Universal Precautions Toolkit, and the AHRQ’s Patient Education Materials Assessment Tool Guide. The methodology was guided by the Interprofessional Shared Decision-Making Model and the AHRQ’s Making Informed Consent an Informed Choice guide. In the preproduction phase, we (1) reviewed the current consent form; (2) observed 18 consent processes; (3) surveyed the vascular access team (N=6 nurses) about their perception of the current process; (4) surveyed 30 patients for knowledge recall and retention, overall satisfaction, and attitude toward using a multimedia PtDA; and (5) wrote and reviewed the script for the multimedia program. The production phase focused on filming the PtDA in English and Spanish languages. The postproduction phase included integrating the multimedia programs into the care processes, developing a modified workflow for the consent process, and alpha testing of the English and Spanish PtDAs by (1) a group of 5 patients for clarity and understandability of the information; (2) nurses using the AHRQ’s Patient Education Materials Assessment Tool Audio and Video; and (3) by the multidisciplinary change team.

**Results:**

Based on the alpha testing, patients indicated that the content was easy to follow and read; nurses provided positive feedback, and their comments were mainly related to the changes in the workflow in the consent process of the PICC after using the PtDA; and the multidisciplinary change team suggested edits related to changing a few scenes. The final multimedia program consisted of 7 min and 37 s demonstrating detailed information about the PICC.

**Conclusions:**

A systematic development of PtDAs for nonurgent invasive procedures may eliminate many limitations of the conventional consent process by ensuring comprehensive, standardized, and easy-to-comprehend information and providing sufficient time for the patients to reflect on the information. To be effective, PtDAs should follow a systematic, patient-centered, evidence-based, and rigorous approach in the development, implementation, and evaluation processes.

**International Registered Report Identifier (IRRID):**

RR1-10.2196/10709

## Introduction

### Overview

Informed consent has considerable clinical, ethical, and legal implications for patient safety, liability, treatment cost and outcomes, patient-centered care, Hospital Consumer Assessment of Healthcare Providers and Systems scores, and reimbursement [1,2]. Patients may sign the consent form without a complete understanding of the indications, benefits, and risks of treatment procedures. A culture of safety that embraces patient engagement in care is required for effective informed consent process. The lack of effective communication between the health care team and the patient is a root cause for informed consent–related sentinel events [2]. The Joint Commission (JC) and the Agency for Healthcare Research and Quality (AHRQ) urged hospitals to provide high-quality decision aids to support the informed consent process [1,2]. This paper describes a systematic approach to develop, implement, and *alpha* test an effective multimedia decision aid to reform the informed consent process of a peripherally inserted central venous catheter (PICC) procedure.

### Background

Patient decision aids (PtDAs) are an integral component of a shared decision-making model. These evidence-based tools help people make informed decisions congruent with their personal values and preferences about their treatment options. Extensive work has been undertaken to test the effectiveness of PtDAs [3-10]. In a 2017 Cochrane review of 105 studies with 31,043 participants, the use of PtDAs helped patients feel clear about their personal values and improved the rate of patient engagement in the decision making by reducing the proportions of undecided patients and passive decision makers compared with usual care [4]. In addition, the use of more detailed PtDAs and those with expressed risk probabilities resulted in a significant knowledge improvement and accurate risk perception compared with basic PtDAs [4].

On the other hand, many of the available trials failed to provide sufficient details about the development process of their PtDAs [11]. Furthermore, little information is available about the process of integrating PtDAs into routine care. The availability of PtDAs in different formats, delivery modes, and information displays, and the varying levels of patient involvement in development and use introduce challenges in the development, delivery, and evaluation of such tools and require detailed description of their development, implementation, and evaluation processes. PtDAs should follow a systematic approach for development to judge the effectiveness of these tools and create reproducible products and replicable methodologies. PtDAs should also include the appropriate level of details about treatment procedures, taking into consideration health literacy principles to help patients make informed choices about their treatments. Standards for patient comprehension and effectiveness measures should also be in place [1,2].

Advances in multimedia technology have increased the utilization of multimedia PtDA programs to supplement the conventional informed consent process that is solely based on a face-to-face discussion. Multimedia is the “field concerned with the computer-controlled integration of text, graphics, drawings, still and moving images (video), animation, audio, and any other media where every type of information can be represented, stored, transmitted and processed digitally” [12]. The majority of the available studies used multimedia tools to support the informed consent process for surgeries and consistently found significant effects on reducing patient anxiety, improving patient understanding of the indications, risks and benefits of the surgery, and increasing satisfaction with the informed consent process [5,9,10,13-17]. Despite these promising results, little information is available about the use of multimedia PtDAs in the consent process of therapeutic invasive procedures such as the PICCs. In addition, none of the available studies have designed their multimedia tools based on the patients’ information needs and the AHRQ’s comprehensive guide for informed consent, *Making Informed Consent an Informed Choice* [1]. To respond to these gaps in the literature, our systematic process of developing, implementing, and evaluating the multimedia PtDA program to reform the informed consent process for the PICC procedure was based on the AHRQ’s guides for informed consent [1], health literacy [18], and patient educational materials [19]; the Interprofessional-Shared Decision-Making (IP-SDM) model [20]; the PtDAs’ quality criteria developed by the International Patient Decision Aid Standards (IPDAS) Collaboration [21]; and the Multimedia Production Framework [22].

### Objective

This paper describes a systematic approach to develop, implement, and *alpha* test a multimedia decision aid to reform the informed consent process of a PICC procedure. PICC is one of the most commonly performed invasive procedures in intensive care units (ICUs) and acute care units (ACUs) and the only invasive procedure where nurses are the responsible clinicians to obtain the consent form. At our hospital, 220 to 250 PICCs are inserted monthly by nurses from the vascular access team—a team of certified nurses for PICC insertion, safety, and care. These catheters are inserted when a prolonged intravenous medication, nutrition or fluids, or blood draw is required.

Our current PICC consent process lacks the use of any decision aids to improve patient engagement in the process [1,2]. In busy ICUs and ACUs, patient acuity and workload can hinder effective patient-health care team member communication and negatively affect providing *informed* consent, especially for the PICC as the most common invasive procedure in these units. The use of a PICC multimedia PtDA in ICUs and ACUs would optimize care outcomes and most importantly, engage patients in care processes by emphasizing the patient role in the safety of the PICC, an area that is often ignored in informed consents.

## Methods

### Overview

**Figure 1:**
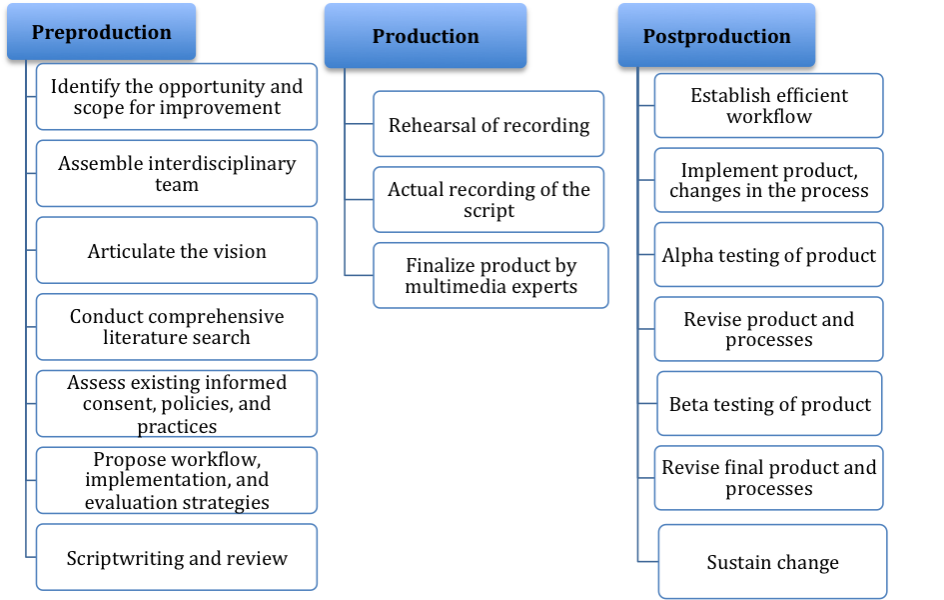
Process of producing, implementing, and evaluating the multimedia decision aid program for a peripherally inserted central catheter.

This quality improvement project was approved by the institutional review boards (IRBs) of the University Health System (setting of the study) and the University of Texas Health at San Antonio, Texas (the institution of the principal investigator) as non-regulated research. After the IRB approval, the multimedia PtDA program was developed for patients undergoing a PICC in the following 10 inpatient units: 2 medical surgical ACUs, 3 surgical ACUs, 1 hematology and oncology unit, 3 surgical ICUs, and 1 medical ICU. The PtDA was created for competent patients and family members or legal guardians of all patients, whether competent or incompetent patients. The development, implementation, and evaluation processes of the PtDA followed the phases described in the Multimedia Production Framework (see Figure 1): preproduction (planning), production (filming), and postproduction (testing and editing) [22]. This paper focuses on the development, implementation, and *alpha* testing processes of the PICC multimedia PtDA. Beta testing of the product (see Figure 1) will be presented elsewhere. Within the Multimedia Production Framework, we applied the Criteria for Judging the Quality of PtDAs developed by the IPDAS Collaboration [21], the AHRQ’s Health Literacy Universal Precautions Toolkit Guide [18], and the AHRQ’s Patient Education Materials Assessment Tool Guide for Audio and Video Materials [19]. The methodology to improve the consent process was guided by the IP-SDM model [20] and the AHRQ’s Making Informed Consent an Informed Choice comprehensive guide for informed consent [1].

The IP-SDM model extends the decision-making process beyond the patient-provider dyad to include the interprofessional team. Patients in ACUs and ICUs usually face decision uncertainty or conflict related to the complexity of their medical conditions and the need for multiple therapeutic procedures. Within an organizational structure and social norms (macro system level), the IP-SDM model captures the complexity of the decision making in the daily organizational operations (meso system level, ie, informed consent process). The model focuses on the patient-family-interprofessional collaboration and places the *patient* at the center to emphasize a patient-centered approach of care [20]. Each individual in the model (ie, the patient, the family, and any member from the health care team) is a micro system [20]. The patient-centered process to make a decision outlined by the model includes the following: (1) understand the decision to be made and explore related options, (2) obtain and share information, (3) clarify one’s own values and preferences, (4) evaluate the feasibility of the decision, (5) select the preferred choice, (6) implement the decision, and (7) assess the outcomes. It is important to note that these 7 activities are not only limited to the patient but also apply to all individuals involved in the shared decision-making process. In this study, the use of a multimedia PtDA for the PICC to supplement the consent process aims to help the patient-interprofessional-family interaction in the first 5 steps in the process.

Consistent with the IP-SDM model, the AHRQ’s guide to reform the informed consent process creates a shared vision about what constitutes an effective informed consent at the macro, meso, and micro system levels and stresses the importance of engaging all stakeholders in the process. From that perspective, challenges to an informed decision might be at the macro (organization), meso (policies and procedures related to informed consent), and micro system levels (individuals in the patient-family-interprofessional team collaboration). For example, in the PICC consent process, challenges to an informed consent might result from lack of a culture of safety at the organization level (macro system), lack of clear policies and procedures for the informed consent, lack of decision support tools (meso level), ineffective patient-interprofessional interaction and communication, lack of family and social support, lack of knowledge about the available treatment options, mismatch between treatment options and the patient values and preference, and the complexity of the medical condition that hinders reaching a *preferred choice* (micro system level).

### Phase 1: Preproduction Phase

Guided by the AHRQ’s comprehensive guide for informed consent [1], this phase focused on identifying the consent process for the PICC as the opportunity for improvement, assembling an interdisciplinary change team with a change authority and a clear vision, agreeing on a plan for change, understanding the limitations of the current consent process, conducting comprehensive literature search, proposing a plan for implementation and evaluation, and writing and reviewing the script for the multimedia program.

#### Step 1: Formulate an Interdisciplinary Team and Articulate the Vision

A multidisciplinary change team was assembled to reform the consent process of the PICC procedure and develop the multimedia PtDA program to supplement the conventional process. The team included bedside nurses from the ACUs, a radiologist, a radiology nurse, nurse educators from the ACUs and ICUs, nurses from the vascular access team, the nursing director of the ACUs, the Vice President and Associate Chief Nursing Officer for Clinical Excellence and Ancillary Services, experts in marketing and communication from the Corporate Communications department, expert nurses from the Office of Patient Experience, experts in multimedia production, Information Technology (IT) department, and experts in health informatics. To sustain the change, the change team members were recruited from different organizational levels to include micro (eg, bedside nurses from the unit), meso (eg, Corporate Communications, IT), and macro system (eg, nurse directors) and had a change lead role and authority to implement a change. Consistent with the hospital values to provide a Safe, Timely, Effective, Efficient, Equitable, and Patient-Centered (in short, STEEEP) care, the team had a shared vision to make the informed consent an informed choice.

#### Step 2: Assess Existing Informed Consent, Policies, and Practices

A review of the current informed consent document and policies was necessary to make sure the information is based on recent practice guidelines for central venous access and health literacy universal precautions and to maintain consistent information across the policies, the consent form, and the multimedia program. We found that our PICC consent form was created 10 years ago with no revision based on new evidence regarding risk factors of the PICC and expressed probabilities of its complications. The form also lacked the definition and details about the procedure. Therefore, a comprehensive literature search was conducted to locate and analyze best practice guidelines for central venous access and credible recent studies [23-33]. All guidelines and studies were summarized in terms of definition of a PICC; possible insertion sites; the need for anesthesia; the need for diagnostic equipment such as ultrasound or x-ray; indications; benefits; expected time period for having the catheter; common, less common, and rare risks and complications; health care team role in the care and safety of a PICC during hospitalization; patient role in the care and safety of a PICC during hospitalization; safety tips when a patient leaves the hospital with a PICC; and other possible treatment options. The research and change team agreed on the final document. Experts from the hospital Corporate Communications department reviewed the summary for fifth grade readability level and clarity based on the AHRQ’s Health Literacy Universal Precautions Toolkit Guide [18], and necessary changes were made. On the basis of this summary, the definition of a PICC, details about the procedure, indications and risk factors, as well as expected complications with their probabilities were added to the consent form.

In addition to examining the consent form, 3 methods were used to understand the limitations of the conventional consent process of a PICC to develop an effective multimedia program. These methods include observing the current process and assessing the perceptions of nurses and patients about the current process. The research team reviewed and approved all tools, checklists, and surveys used for this purpose.

##### Observing the Consent Process

First, we observed 18 informed consent processes for the PICC procedure provided by the vascular access team nurses (N=6 nurses, 3 observations per each nurse). Two nurse educators independently conducted the observations using a standardized checklist to enhance objectivity. Observations included language of the discussion, time spent by the vascular access team nurse to provide patients information about the PICC procedure and sign the consent form, speed of discussing the information, level of distraction during the discussion, patient level of discomfort, type and adequacy of the content discussed by the vascular access team nurse, and questions asked by the patient during and after the discussion. The observers used the summary document created by the research and change team in Step 1 to guide their observations with regard to the content discussed. The 2 observers met after each observation session to review similarities and differences and reach consensus. Below is a summary of the observation results.

- Language: All consent processes were discussed in English language based on the patients’ preferred language.
- Time: Nurses from the vascular access team spent on average 2 to 7 min discussing the procedure to the patient and obtaining the signature on the consent form, with a mean of 4.6 min (SD 1.4). Out of 18, 7 procedures took 2 to 3 min of discussion.
- Distraction: The observers reported 8 (out of 18) processes with distraction because the television was switched on, the patient’s room door was open, or the patient received a phone call during the discussion.
- Patient comfort level: According to the observers, only 1 patient did not look comfortable (ie, had pain) during the discussion.
- Speed of the discussion: The observers reported *very fast* as the speed of discussing 4 (out of 18) observations. All other observations demonstrated appropriate speed.
- Information provided and adequacy of information: The 2 observers assigned *adequate* to all content, when discussed. Table 1 summarizes the number of episodes where nurses from the vascular access team did not discuss the content.

Questions asked by the patients during the discussion included:

1. Will I feel the needle going in? (1 patient)
2. Why you will have a mask? (2 patients)
3. Will I be covered up? (1 patient)
4. How long will the vein hurt? (1 patient)
5. Do I have to stop eating? (1 patient)
6. Will I get a CAT scan? (1 patient)
7. I am left-handed; do we have to go in on the left arm? (1 patient)
8. Are you going to take x-rays before or after? (1 patient)
9. Do I need to remove my necklace? (1 patient)

Questions asked by the patients after the discussion included:

1. Is this going home with me? (1 patient)
2. How to keep the line from coming out and after caught up on things? (1 patient)
3. What kind of line is this? What is this supposed to do? (2 patients)
4. Do I have to take antibiotic pills? (1 patient)
5. How long will I have this? (3 patients)
6. Will I have the same one throughout the chemotherapy? (1 patient)
7. What are the side effects of this procedure? (1 patient)
8. Who can remove it? (1 patient)
9. Why are my veins so small? Is it because I am on blood thinners? (1 patient)
10. So you’ll check it with the x-ray after? (1 patient)

**Table 1:** Number of consent processes when the content was not discussed (N=18 observations).

Content	Number not discussed, n (%)
Definition of a PICC^a^	0 (0)
The need for anesthesia	0 (0)
Steps of the procedure itself (preparation, during, and after the procedure)	0 (0)
The need for diagnostic equipment such as ultrasound or x-ray	1 (5)
Indications or reasons	1 (5)
Benefits	1 (5)
Common or less common and rare risks and complications	1 (5)
Verification of patient and family members’ understanding of the procedure	1 (5)
Possible insertion sites	2 (11)
Expected period for having the catheter	8 (44)
Health care team role in care and safety of a PICC^a^ during hospitalization	16 (89)
Other treatment options	16 (89)
Patient role in care and safety of a PICC^a^ during hospitalization	17 (94)
Safety issues when a patient leaves the hospital with a PICC^a^	18 (100)

^a^PICC: peripherally inserted central catheter.

The results of the observations identified the limitations of the conventional process. For example, spending 2 to 3 min in the discussion and obtaining the consent form does not reflect an effective informed consent process with a teach-back mechanism. Many of the consent processes also lacked discussing critical points (ie, patients and health care team role in the safety of a PICC). On the other hand, it is important to note that some of the questions asked by the patients after the discussion were already discussed by the vascular access team nurse during the consent process (ie, What kind of line is this? What is this supposed to do? What are the side effects of this procedure?). These results support the need for a reliable multimedia PtDA to supplement the conventional process to allow patients to view the content as many times as needed and when they are ready to do so (ie, no distraction and no pain). The program should include necessary details about the procedure and be recorded with an appropriate speed of presenting the information.

##### Nurses’ Perceptions About the Current Process

Second, we surveyed all nurses from the vascular access team (N=6) about the limitations of the current PICC consent process and suggestions for improvement. Nurses from the vascular access team are males with 3 to 17 years of experience in placing and maintaining PICC lines. All nurses have a bachelor’s degree in nursing science and 1 nurse has an advanced degree as a nurse practitioner. Major issues nurses faced in providing informed consent for a PICC were related to time constraints. When asked about their opinion to use the multimedia to supplement the consent process, nurses responded very favorably emphasizing that the tool should be used to supplement rather than to replace the discussion between the patients and the health care team members.

##### Patients’ Perceptions About the Current Process

Third, we surveyed 30 patients who received the consent process for the PICC procedure for knowledge recall and knowledge retention about the procedure, overall patient satisfaction with the consent process, and attitude toward using a multimedia PtDA to supplement the consent process. Corporate Communications reviewed and approved the final versions of all patients’ surveys for clarity and readability. Moreover, 2 nurse educators administered the surveys. The medical record number was used to connect patients’ responses on all questionnaires. Out of the 30 patients, 53% (16/30) reported their level of education as high school and the other 47% (14/30) reported college or graduate studies. The sample also included 10 (33%, 10/30) male patients and 20 (67%, 20/30) females. Ethnicity was almost equally distributed among non-Hispanic white (47%, 14/30) and Hispanic patients (40%, 12/30). Moreover, 4 patients (13%, 4/30) were black. Patients were selected from the 10 patient units where a PICC was inserted. A description of the questionnaires and the results are presented in Multimedia Appendix 1.

The questionnaire used for knowledge recall and knowledge retention was created based on recent guidelines for PICC [23-33] and included 3 *select one-answer* multiple-choice questions, 4 *select all that apply* multiple-choice questions, and 12 *true or false* questions. The knowledge recall questionnaire was administered within 4 to 8 hours after the discussion between the vascular access team nurse and the patient and obtaining the signed consent. The same questionnaire was administered to the same patients 24 to 48 hours after the consent process to measure knowledge retention. Multimedia Appendix 1 describes the percentages of correct answers selected by the patients. The correct responses are indicated for the multiple-choice questions and identified at the end of the question for true or false questions (see Multimedia Appendix 1, column 1). The mean score of knowledge recall was significantly lower than the knowledge retention (mean 12.6, SD 2.27, compared with mean 14.57, SD 1.9, respectively, paired sample *t* test=3.6, *P*<.001).

Out of the 19 knowledge questions, only 2 items were answered correctly by all patients in the knowledge recall and 3 in the knowledge retention questionnaires (see Multimedia Appendix 1). For ethical purposes, the data collectors corrected the wrong answers provided by the patients in the knowledge recall questionnaire after recording the original responses provided by the patients. This could explain the higher knowledge retention scores in comparison with knowledge recall. Items with the lowest knowledge recall and knowledge retention scores were related to common and rare risks of a PICC (Items 4 and 5), signs of infection from a PICC that a patient should report (Item 6), if the patient can move around freely (Item 8), and the frequency of inspecting the line site by the nurse (Item 9).

Patient satisfaction with the informed consent process consisted of 9 items with a 5-point Likert-type scale of agreement and was created based on the essential elements emphasized in the AHRQ’s comprehensive guide for informed consent [1]. At the end of the survey, patients were asked to report their overall level of satisfaction with the informed consent process using a 5-point Likert-type scale that ranged from 5=very satisfied to 1=very unsatisfied, to write any additional comments about the information they received and to write other information they would like to know about the procedure to provide an informed consent. The survey was administered 4 to 8 hours after the consent process.

Table 2 shows the results of the patient satisfaction with the informed consent process. Patients’ responses were coded as Agree for strongly agree and agree and as Disagree for strongly disagree and disagree. Neutral responses remained neutral in the analysis.

Out of the 30 patients, 27 (90%, 27/30) agreed that the information provided was comprehensive. Missing content reported by patients were other treatment options, provider role in care and safety of a PICC (10%, 3/30), and patient role in the care and safety of a PICC (7%, 2/30).

The mean patient satisfaction score with the PICC consent process using a 5-point satisfaction scale was 4.8 (SD 0.37). Patients added that they would like to know more about their role in the PICC (7%, 2/30) and to involve their families in the consent process (7%, 2/30).

In summary, all patients were satisfied with the consent process and felt that the timing of the discussion was convenient. Inconsistent with the knowledge recall scores, all patients reported that they completely understand the common complications of this procedure. Inconsistent with the observers’ ratings of the process, all patients felt that the speed of discussing the information was reasonable. Only 2 (7%, 2/30) patients disagreed to the item “I understand my role as a patient in maintaining the safety of the PICC line.”

In addition to patient satisfaction, patients’ attitudes toward using a multimedia PtDA program to supplement the consent process survey was created based on the main benefits of using multimedia PtDAs identified in the literature [5,9,10,13-17]. The survey consisted of 6 items of a 5-point Likert-type agreement scale where 5=strongly agree and 1=strongly disagree. In this survey, we used the term *video* instead of multimedia to enhance the readability and understandability of the items by the patients. The survey was administered at the same time of administering the patient satisfaction survey. Patients’ responses were coded as Agree for strongly agree and agree and as Disagree for strongly disagree and disagree. Neutral responses remained neutral in the analysis. As shown in Table 3, patients reported a high positive attitude toward the use of multimedia as a supplement to the conventional process.

In addition to examining the current process, at this stage, a comprehensive literature search for the use of multimedia as a supplement to the conventional informed consent process was conducted. The research and change team also proposed the method for implementing and evaluating the multimedia PtDA and discussed the changes in the workflow of the PICC consent process. Details about the implementation and changes in the workflow are described in the postproduction phase.

#### Step 3: Scriptwriting

The principal investigator created the first draft of the script based on the PICC guidelines, the procedure content identified in Step 1, the limitations of the current process, the results of the patients’ and nurses’ perceptions about the current process, the first 5 activities of the patient-centered process to make a decision outlined by the IP-SDM model, the AHRQ’s comprehensive guide for informed consent [1], the AHRQ’s Health Literacy Universal Precautions Toolkit Guide [18], and the AHRQ’s Patient Education Materials Assessment Tool Guide for Audio and Video Materials [19]. The script included introduction; purpose of the educational program; intended users of the program; disclaimer; background including a definition of the PICC and possible insertion sites; purpose of the line; other treatment options; common, less common, and rare risks and complications; education; procedure (before, during, and after, including the need for diagnostic equipment such as ultrasound or x-ray); expected time period for having the catheter; safety issues when a patient leaves the hospital with a PICC; and a conclusion. The introduction encouraged patients and family members to write down questions they might have about the procedure. The conclusion emphasized the need to ask all questions before signing the consent form. There was a great emphasis in the script on the health care team and patient roles in the care and safety of a PICC during hospitalization. The content stressed on clarifying the patient’s values when considering the decision, that is, “Your provider discussed with you all other treatment alternatives of a PICC. Please let us know if you feel you need further information about these alternatives” and “Please make sure that you understand the risks, benefits and complications of the procedure before you sign the consent form.”

The provider usually discusses other treatment options with the patient before ordering the PICC line, the details of these treatment options, and associated risks. Treatment options are individualized based on the patient condition and the reasons for the PICC and may include having another type of central line, for example, implanted port in the chest wall, whether an acceptable substitute for a PICC or changing the medication, and whether the intended infusion is a medication that has a potential to damage the peripheral veins. After explaining the intended treatment plan and all available options to the patient, they should be given the opportunity to agree or disagree with the plan of care before the provider places a PICC order in the electronic medical record (EMR). If the patient has questions about an alternative to the PICC placement, those questions are referred to the provider. Nurses from the vascular access team usually accompany the provider in this discussion. However, they are not qualified to discuss the risks and benefits of the alternative treatment options with the patient directly; they can explain the details of the PICC procedure.

**Table 2:** Patient satisfaction with the informed consent process (N=30).

Item	Agree, n (%)	Neutral, n (%)	Disagree, n (%)
The information provided was clear	30 (100)	0 (0)	0 (0)
The information provided was easy to understand	30 (100)	0 (0)	0 (0)
Timing of the discussion was convenient	30 (100)	0 (0)	0 (0)
Speed of information provided was reasonable	30 (100)	0 (0)	0 (0)
Provider attitude was positive during the discussion session	30 (100)	0 (0)	0 (0)
I completely understand the common complications of this procedure and know when to report them	30 (100)	0 (0)	0 (0)
Disruption during the discussion was minimal	29 (97)	1 (3)	0 (0)
I understand my role as a patient in maintaining the safety of the PICC^a^	28 (93)	0 (0)	2 (7)
The information provided was comprehensive to include: definition of the PICC; reasons for the PICC; steps of the procedure; common side effects; other treatment options; patient role in care and safety of the PICC; provider role in care and safety of the PICC; if the provided information was not comprehensive, please circle the missing content from the contents above	27 (90)	1 (3)	2 (7)

^a^PICC: peripherally inserted central catheter.

**Table 3:** Patient attitude toward using a multimedia program to supplement the consent process (N=30 patients).

Item	Agree, n (%)	Neutral, n (%)	Disagree, n (%)
I think the use of a recorded video about the procedure would be beneficial	29 (97)	1 (3)	0 (0)
The use of the video will allow patients to listen to the information as much as they need	29 (97)	1 (3)	0 (0)
The video will allow a patient to listen to the information about the procedure when he or she is ready to do so	29 (97)	1 (3)	0 (0)
The use of the video will better help a patient recall the information about this procedure	29 (97)	1 (3)	0 (0)
The video will decrease the patient level of anxiety	29 (97)	1 (3)	0 (0)
I highly recommend the use of the video as a supplement to the consent process	26 (87)	3 (10)	1 (3)

The script went through a review process by all members of the multidisciplinary change team. This process proved to be the most time consuming. Multiple versions of the draft were revised to lower the reading level. Some considerations in scripting included:

- deciding on the narrator and persons in the scenes
- determining the types of the scenes including the background of shooting, recording setting, the equipment, and pictures to be shown such as, the vascular access team picture, hospital logo, and the anatomy of the vascular system connecting to the heart where the PICC line will be inserted and the catheter tip location
- developing bullet points for the worded graphic slides to help patients understand the important concepts
- determining the sequences of the scenes (ie, sequence between the narrator, the worded graphic slides, the pictures, the patient room, etc).

After validating the final version of the script by the research team, 10 patients reviewed the script for clarity and readability. Among the 10 patients, 5 were male, 6 were Hispanic, and 7 indicated their educational level as high school, whereas 3 reported college degree. Patients’ comments were related to clarifying the terminologies in the complications section of a PICC, such as *blood stream infection* and *deep tissues* and *embolism* in the following statement:

If there is damage to the catheter and the surrounding deep body tissues, a blood clot could travel into the blood to the lungs and cause pulmonary embolism.

The statements were clarified and sent back to the same patients who indicated that the statements look much easier to understand after the modifications.

A certified company (MasterWord Services) translated the English script into Spanish for our Spanish-speaking patient population to ensure medically accurate translation of the script. Moreover, 2 expert nurses from the vascular access team were approached to voluntarily serve as the narrators. English was the mother tongue of the nurse who volunteered to record the English version of the program, and Spanish was the mother tongue of the nurse who volunteered to record the Spanish version of the program. The change team also asked a nurse to play a patient role during the recording of the vascular access team-patient encounter to maintain patient privacy.

### Phase 2: Production Phase

The hospital contracts with a local videographer and photographer to produce videos and photography projects. The team created still photography and developed multiple worded graphic slides to emphasize specific messages that we wanted the patients to remember to improve patient retention of material. These slides allow points where patients can pause the video and better understand warning or complication signs, their role in care processes, and other treatment options.

The actual production started with a rehearsal of the recording by the narrators and the multimedia experts. Different team members were present to provide feedback. Multimedia experts recorded the video in a variety of daylong shoots over several months using a Blackmagic 2.5K cinema camera. The recording took place in our hospital system video and photography studio and in a patient room where a bedside nurse played the role of a patient. Experts used professional lighting to help decrease the glare from the typical room lighting and made use of a green screen backdrop that allowed the research team to drop in neutral background images during the narration scenes.

The video shooting required multiple angle shots to provide us with options to tell the story of a PICC line insertion. Multiple video B-roll shots gave us alternatives to best display the nurse and patient interaction as well as close-up shots of the insertion, washing hands, sterile gowning, and an example of the PICC line insertion. A Shutterstock graphic showed veins and the heart to allow patients to see on a line-drawn image exactly where the PICC line would enter the vein and how it would approach the heart. A close-up photo of the PICC line not only allowed our video editing team to cover a cut between camera angles but also gave an opportunity to show the patient exactly what the line will look like. We also showed the consent form interaction exactly as it should happen, with the patient signing on the tablet.

**Figure 2:**
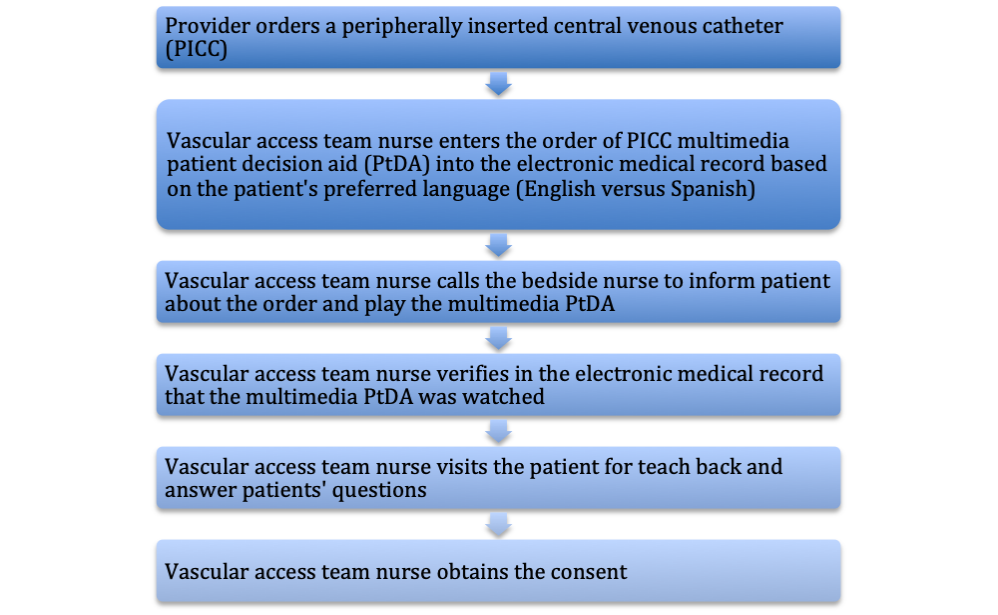
A modified workflow of the peripherally inserted central venous catheter (PICC) informed consent process.

### Phase 3: Postproduction Phase

The postproduction phase included integrating the multimedia PtDA program into the care processes, approval of the modified workflow of the PICC consent process, and alpha testing of the product. The IT department integrated the multimedia program into the Interactive Patient Care solution GetWell Inpatient. This solution is a personalized patient education and entrainment system that includes many videos and multimedia programs and is connected to the EMR for the purpose to engage patients and their families in their care. Patients may also use the system to provide feedback through various surveys, request housekeeping services, or to share their experience with patient relations. In addition, patients have access to a variety of television channels and recently released box office movies. All health education videos and multimedia programs within this system that are assigned through an order and watched by the patients are recorded in the EMR along with the time and frequency of watching.

The PICC multimedia program was added to the GetWell Inpatient Health Education Library with an ancillary code that was also built in the EMR on the patient education order. Health level 7 (HL7) admission, transfer, and discharge messages were sent to GetWell Inpatient through the Cloverleaf Interface Engine. The patient unit, room, and bed location in the admission, transfer, and discharge message were stored in the Interactive GetWell Inpatient system. The PICC PtDA multimedia program was embedded into the PICC order set within the EMR. When a PICC is ordered, the PICC education is ordered automatically and sent via HL7 order result message to the GetWell Inpatient system in the patient’s room. This will lead to a notification display on the room television notifying the patient that an educational item has been ordered. When the education video has been viewed, an HL7 observation result message is sent back to the EMR with the time viewed and closes the education order. The EMR medical logic module writes all education results to the patient education log as well as to the results section of the EMR. After the integration of the multimedia program into the GetWell Inpatient, the change team approved the new workflow of informed consent as a result of using the PtDA program (see Figure 2).

In addition to the multimedia program, the research team also created an information sheet that includes the same information presented in the program about the PICC. The information sheet provided another resource for competent patients and family members.

The English and Spanish multimedia programs integrated into the EMR went through 3 alpha testing or validation phases. First, each program was tested by a group of 5 patients for ease and clarity of the language, understandability of the information related to the procedure, readability of the font when slides were displayed within the video, and clarity of the critical points. Test patients were selected from different educational levels and genders. Second, the programs were reviewed by all nurses from the vascular access team and 7 bedside nurses for logical sequence of the discussion, quality of the scenes, and adequacy of the information. Nurses used the AHRQ’s Patient Education Materials Assessment Tool Audio and Video to rate the program [19]. The tool consists of 17 items—13 for understandability and 4 for actionability [19]. Third, the multidisciplinary change team reviewed the final product and for the second time applied the Criteria for Judging the Quality of PtDAs developed by the IPDAS Collaboration [21].

## Results

Based on the alpha test, (1) the test patients indicated that the content of the programs was easy to follow and understand, and the font was readable; (2) all nurses provided positive feedback and their comments were mainly related to the changes in the workflow in the consent process of the PICC after using the PtDA; and (3) the suggested edits by the change team were related to changing few scenes (ie, a scene related to a nurse who played a family member role touching the PICC line). The final multimedia program consisted of 7 min and 37 s demonstrating detailed information about the PICC.

After the alpha test, the research and change teams created a plan to train all nurses from the vascular access team and bedside nurses on the new workflow to standardize the informed consent process. Two nurse champions were selected from each of the 10 ACUs and the ICUs to facilitate the training.

## Discussion

### Summary of Protocol Findings

This paper described a multidisciplinary, patient-centered, systematic process to develop, implement, and alpha test a multimedia PtDA program to reform the consent process of the PICC procedure. Our development, implementation, and evaluation processes were based on the IP-SDM model [20], the AHRQ’s national guides for informed consent and health literacy [1,18], PtDAs’ quality criteria developed by the IPDAS Collaboration [21], and the Multimedia Production Framework [22]. We are currently in the process of beta testing the program and will publish the results in the near future. The beta testing will focus on implementing the program and the effect of the PICC PtDA program on patient knowledge recall, knowledge retention, satisfaction with the multimedia program, and satisfaction with the informed consent process.

The preproduction assessment phase of this study identified the limitations of the conventional PICC informed consent process and supported the need for a PtDA to supplement the consent process [10]. Main limitations were related to the use of an outdated consent form that also did not include necessary information about the procedure definition, steps of the procedure, complications, and patient role in the safety of a PICC. Observing the current informed consent process revealed spending a short period in the process; availability of distraction during the discussion; not considering the patient comfort level, which may affect patient readiness to engage in a discussion; and inadequacy of the information discussed with the patients. Almost 90% to 95% of the observed processes missed discussing the health care team role and the patient role in the care and safety of a PICC during hospitalization. During the observations, we also found that patients ask questions about content that was already discussed by the vascular access team during the consent process. This may (1) reflect the shortcomings of the current process (ie, workload-related factors such as not spending enough time to discuss the procedure with the patient, or patient-related factors that affect comprehension such as pain and discomfort), (2) support the need for a self-paced resource such as multimedia PtDA available to patients when needed, or (3) suggest that the medical conditions of some patients in the ICUs and ACUs might be a barrier for patient engagement in the consent process [34], which advocates for the need to engage a proxy in the informed consent process [35].

Although patients were satisfied with the current consent process and believed they have sufficient knowledge about the procedure, associated risks and their role in the safety of the procedure and risks and complications about the procedure were the items with the lowest scores in the knowledge recall questionnaire. These results further support the need for a self-paced tool to better inform patients about the procedure.

Multimedia PtDA tools can be delivered to patients using DVDs, iPads, or directing the patient to an authenticated or unauthenticated website or patient portal where the information is stored on the health care setting internet or intranet [36]. In this study, we delivered the program using the Interactive Patient Care solution GetWell Inpatient. The system is integrated into our workflow since 2014 and is used to push many educational videos and multimedia programs to patients in addition to other purposes. The main benefits of using GetWell Inpatient are the ability of the patients to review the information at the point of care, when needed, as many times as needed, and to engage their families in the process, and the ability of the EMR to track the use of educational videos and multimedia programs by the patients.

Multimedia PtDAs are effective tools to engage patients in care processes and treatment options. Cost is one of the factors that may limit the production and utilization of multimedia PtDAs [3]. The costs associated with producing a multimedia PtDA vary with some contributing factors to include availability of expertise, video length, recording location (hospital environment versus studio), number of still images, and additional graphic enhancements. The filming time of our PICC PtDA (setting up, lighting, shooting, breaking down, etc) for both the English and Spanish versions to include B-roll was a total of 14.5 hours. Pre- and filming time periods were approximately 30 to 40 hours. The total cost associated with filming the PICC PtDA was approximately US $7800. The entire project took 15 months to complete, from January 2017, to March 2018.

### Implications

The methodology we used to reform the consent process of the PICC includes essential steps that can be used to reform the informed consent process for any therapeutic procedure in other health care settings. These steps include:

- assembling a multidisciplinary change team with a change in authority
- articulating a clear vision
- conducting a comprehensive literature search about the procedure and multimedia apps
- rigorous assessment of the limitations of the current informed consent process, policies, and practices
- writing a script that is based on national guides, limitations of current process, and recent literature
- engaging patients in the evaluation of the current process and proposed change
- engaging all clinicians responsible for obtaining the consent for the procedure
- using credible guides and frameworks to guide the process
- using credible tools (ie, AHRQ’s Patient Education Materials Assessment Tool Audio and Video) for assessment
- maintaining consistency across the informed consent document, multimedia app, and policies.

The evaluation process we used was robust and comprehensive to include observing the current process, assessing the perceptions of the vascular access team members about the limitations of the current process, assessing patients’ satisfaction with the current process and their attitude toward the use of multimedia programs, and evaluating patients’ level of knowledge recall and retention about the procedure. The group of patients who was engaged in the evaluation process was from different genders, educational levels, and race or ethnic groups.

Many factors may support the success of the beta testing of our program in the future. First, the improvement opportunity to make the informed consent an informed choice was a priority that was supported by the organization leadership. Second, all stakeholders (leaders, clinicians, and patients) were engaged in the change process. Third, the hospital has sufficient infrastructure for multimedia production. Fourth, GetWell Inpatient allowed seamless integration of the program and tracking of its use. Finally, our PtDA empowered patients by highlighting patients’ role in the safety and care of a PICC line.

The JC requires informed consent to be clear, comprehensive, and engaging. Time pressure is a major challenge to provide an informed consent. Well-designed PtDAs empower patients to make informed decision about treatment options; reduce variation in practice; standardize the amount, quality, and clarity of the information provided; and provide critically ill patients the choice to review the information at times convenient to them, taking into consideration their readiness to learn.

### Limitations

Although we followed a rigorous method to assess the need to reform the PICC consent process and to create and alpha test the PICC PtDA, there are some limitations that need to be considered. First, our patient sample used in the preproduction phase was limited to 30. Engaging more patients might provide additional insight into the limitations of the current PICC consent process that need to be considered when reforming the process. Second, although ethnicity was almost equally distributed among non-Hispanic and Hispanic patients for all patients who participated in the preproduction assessment and those who reviewed the script, the patients who reviewed the script indicated English as their preferred language and therefore reviewed the English version of the program. The beta testing may reveal additional insight for the Spanish version of the program. Third, the IP-SDM model emphasizes the role of family in the decision-making process; however, only few family members were engaged in the initial assessment of this process because many were not available. Some of our findings suggest engaging proxy or family members to help patients in the decision-making process in ICUs and ACUs.

### Conclusions

PtDAs are recommended tools to supplement the informed consent process for treatment procedures. A systematic development of PtDAs for nonurgent invasive procedures can eliminate many limitations in the conventional consent process by ensuring comprehensive, standardized, and easy-to-comprehend information about the procedure and treatment options and by providing sufficient time for the patients to reflect on the information. To be effective, multimedia PtDAs should follow a systematic, evidence-based, and rigorous approach in the development, implementation, and evaluation processes. Including key stakeholders such as leaders, clinicians, and patients is fundamental for the success of these tools.

## Appendix

Multimedia Appendix 1 Number of patients who correctly answered questions in the knowledge recall and knowledge retention questionnaires (N=30 patients).

## Abbreviations

ACUs: acute care units
AHRQ: Agency for Healthcare Research and Quality
EMR: electronic medical record
HL7: health level 7
ICUs: intensive care units
IPDAS: International Patient Decision Aid Standards
IP-SDM: Interprofessional-Shared Decision-Making
IRB: institutional review board
IT: information technology
JC: Joint Commission
PICC: peripherally inserted central venous catheter
PtDA: patient decision aids
