# Supplementary material for: Developing, Implementing, and Evaluating a Multimedia Patient Decision Aid Program to Reform the Informed Consent Process of a Peripherally Inserted Central Venous Catheter Procedure: Protocol for Quality Improvement
Source: JMIR Res Protoc. 2018 Dec 18;7(12):e10709. doi: 10.2196/10709 (PMC6315220; doi:10.2196/10709)
Supplement: Multimedia Appendix 1 [file resprot_v7i12e10709_app1.pdf]

Multimedia Appendix 1. Number of patients who correctly answered questions in the knowledge recall and knowledge retention questionnaires (N=30 patients).

Question	Knowledge recall, n (%)	Knowledge retention, n (%)
Questions: Select one answer		
1. What you need to tell your healthcare provider BEFORE the procedure:<br>A. If you have a tattoo on the site of catheter insertion<br>B. If you had a PICC[a] inserted before this hospitalization<br>C. If you are currently using drugs that thin your blood such as Aspirin (correct answer)	17 (57)	22 (73)
2. A PICC[a] is a thin line placed in your:<br>A. Arm and goes near the heart (correct answer)<br>B. Neck and goes into your heart<br>C. Right arm and goes into your heart and lungs	24 (80)	28 (93)
3. A PICC[a] is mainly used to:<br>A. Improve your urinary output<br>B. Decrease your mobility to maximize comfort<br>C. Draw blood and administer intravenous fluids (correct answer)	29 (97)	29 (97)
Questions: Select all that apply		
4. COMMON risks that you may expect from a PICC[a] are:<br>A. Irregular heart rate<br>B. Tingling in arm or hand (correct answer)<br>C. Minor pain or bruising at the insertion site (correct answer)	8 (27)	9 (30)
5. RARE risks of a PICC[a] are:<br>A. Infection in the catheter (correct answer)<br>B. Inflammation of the deep veins (correct answer)<br>C. Infection at the skin puncture/insertion site (correct answer)	12 (40)	17 (57)

6. The followings are signs of infection from a PICC[a] and should be reported immediately to your healthcare provider:<br>A. Fever (correct answer)<br>B. Headache<br>C. Redness on the skin around the catheter (correct answer)	14 (47)	16 (53)
7. MAIN benefits for a PICC[a] are:<br>A. Allows administration of medicines that irritate small veins (correct answer)<br>B. Eliminate frequent poking of your veins for lab draws (correct answer)<br>C. Eliminate the use of many other peripheral cannulas into small veins (correct answer)	18 (60)	22 (73)
True/false questions		
8. When you have a PICC[a], you can move around freely because the PICC[a] line is sutured (False)	8 (27)	9 (30)
9. Inspection of the line site by the nurse is required once every WEEK to make sure there is no bleeding/misplacement (False)	11 (37)	15 (50)
10. All patients should expect to have the PICC[a] in place for at least 3 weeks (False)	18 (60)	20 (67)
11. A GENERAL anesthesia with ultrasound is necessary to place a PICC[a] line (False)	20 (67)	26 (87)
12. Once a [a]PICC line is in place it is normal to feel minor pain in the area of the insertion for a short period of time (True)	21 (70)	29 (97)
13. When you have a PICC[a], you can NOT take a shower because the dressing should NOT get wet (False)	21 (70)	28 (93)
14. A PICC[a] dressing should be changed daily (False)	23 (77)	27 (90)
15. The nurse can check your blood pressure over the PICC[a] line (False)	24 (80)	28 (93)

16. The need for a PICC[a] should be evaluated daily by your healthcare team members (True)	27 (90)	30 (100)
17. A high fever after a PICC[a] insertion is NOT normal and should be reported (True)	28 (93)	30 (100)
18. During the insertion of a PICC[a], you need to stay still and breathe normally (True)	30 (100)	30 (100)
19. The date of last dressing change should be documented on your PICC[a] dressing (True)	30 (100)	28 (93)

[a]PICC: peripherally inserted central catheter.
